# Neurocognitive Profile and ^18^ F-Fluorodeoxyglucose Positron Emission Tomography Brain Imaging Correlation in Children with Electrical Status Epilepticus during Sleep

**DOI:** 10.1055/s-0042-1757284

**Published:** 2023-06-27

**Authors:** Madhur K. Srivastava, Afshan J. Shaik, Sireesha Yareeda, Kavitha Nallapareddy, Lokesh Lingappa, Pallavi Moturi, Padmaja Gaddamonugu, Rukmini M. Kandadai, Rupam Borgohain

**Affiliations:** 1 Department of Nuclear Medicine, Nizam's Institute of Medical Sciences (NIMS), Panjagutta, Hyderabad, Telangana, India; 2 Department of Neurology, Nizam's Institute of Medical Sciences (NIMS), Panjagutta, Hyderabad, Telangana, India; 3 Department of Pediatric Neurology, Rainbow Children Hospital, Banjara Hills, Hyderabad, Telangana, India

**Keywords:** absolute asymmetry index, CBCL, CSWS, ESES, ^18^ F FDG PET-CT, thalamic hypometabolism, Weschler's scale

## Abstract

**Objective** Electrical status epilepticus in sleep (ESES) is defined by near-continuous epileptiform discharges during sleep along with cognitive, behavioral, and/or imaging abnormalities. We studied the neurocognitive profile and their correlation with ^18^ F fluorodeoxyglucose positron emission tomography (FDG PET) brain abnormalities in children with ESES.

**Methods** Fourteen children with ESES with normal magnetic resonance imaging (MRI) from March to December 2019 were included. The intelligence quotient (IQ) and child behavior checklist (CBCL) scores were estimated using validated scales, and FDG PET brain was done at the same point of time to look for cerebral metabolic defects which was compared with a control group.

**Results** Fourteen patients with a mean age of 8.2 ± 2.7 years were analyzed. The average duration of epilepsy was 6 ± 2.8 years. The mean IQ was 72.4 ± 18.2 and mean CBCL score was 37.3 ± 11.8. There was negative correlation between IQ and CBCL ( *r* = −0.55, *p* < 0.001). The duration of epilepsy also showed negative correlation with IQ ( *r* = −4.75, *p* < 0.001). FDG PET scan showed predominant thalamic hypometabolism in 12 of 14 patients (85.7%) on visual analysis with multiple other hypometabolic cortical and subcortical regions in the brain. The quantitative analysis showed significant difference in metabolism of basal ganglion when compared with control group. The total number of hypometabolic regions seen in the brain showed moderate positive correlation with CBCL score but no significant correlation with the IQ of cases.

**Conclusion** This study demonstrates functional impairment of cerebral cortical, basal ganglia, and thalamic hypometabolism in a cohort of ESES patients with normal structural MRI brain study. There was a moderate correlation of extent and pattern of cerebral hypometabolism with the neuropsychological status of the child and duration of epilepsy.

## Introduction

Electrical status epilepticus in sleep (ESES) is described as spike and wave discharges comprising at least 85% of non–rapid eye movement (NREM) sleep and, since then, it has intrigued the neurologists with its characteristic presence in sleep, lack of proper imaging characteristics, and difficult management of these patients. 1 Even more than 50% of NREM sleep showing electrical activity is enough to classify it as ESES. 2 3 It is a rare disorder with an incidence of 0.5 to 2% of all childhood epilepsies 3 and includes a spectrum of conditions which ranges from mild cases of benign rolandic epilepsy to severe condition like continuous spike and wave in slow sleep (CSWS) with intractable seizures and profound cognitive disturbances. ESES serves as a model for cognitive impairment secondary to prolonged interictal epileptiform activity 4 which is thought to be the culprit for neuropsychological abnormalities observed in these patients. 5 The final neuropsychological outcome of the child is correlated to the duration of ESES 6 and majority of ESES children have persistent intellectual disabilities. 7 8

The pathophysiological mechanisms underlying ESES are less well understood. The most accepted hypothesis is an activation of pathological corticothalamic circuits causing abnormal electrical activation. 9 We tried to study the functional abnormalities in the brain using FDG PET in these children and compared them with age-matched controls. We analyzed whether there is any correlation between the neurocognitive profile and functional abnormalities on PET scans.

## Materials and Methods

It was a prospective cross-sectional observational study, performed from March to December 2019 at a tertiary care hospital in South India. The study was approved by the institutional ethical committee.

## Cases

We prospectively included pediatric patients of 2 to 16 years' age group who presented with a history of at least one episode of seizure and fulfilled the criteria for CSWS as having neurocognitive decline, and overnight sleep electroencephalography (EEG) showing spike and wave discharges in 85% or more of slow-wave sleep. The patients had normal magnetic resonance imaging (MRI) brain study with no structural abnormality. Those patients who had structural abnormality on MRI study or children with a grossly delayed milestone (motor or social) before the onset of CSWS and those children whose parents/guardian refuse to give consent for the study were excluded from the study.

**Fig. 1:**
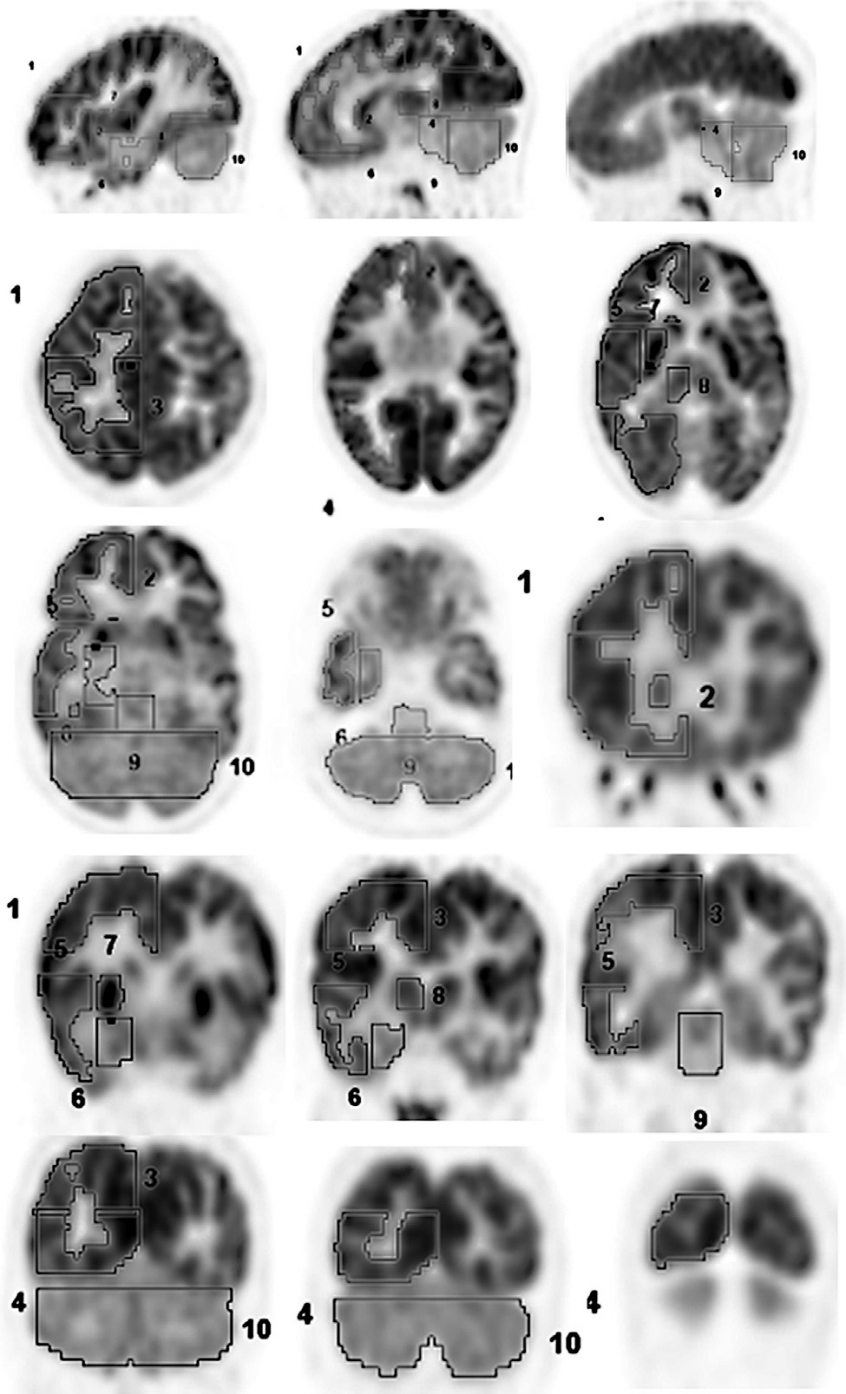
Brain cortical, subcortical, and whole cerebellum ROIs drawn on right side. Similar ROIs were drawn on left side. The maximum FDG uptake in the voxel in ROI depicted as SUVmax was recorded. The various regions are as follows: 1 and 2. right frontal cortex, 3. right parietal cortex, 4. occipital cortex, 5. lateral temporal cortex, 6. right mesial temporal cortex, 7. right lentiform nuclei, 8. right thalamus, 9. midbrain, 10. cerebellum (not depicted – right caudate nuclei). FDG, fluorodeoxyglucose; ROI, region of interest; SUV, standardized uptake value.

The demographic data and clinical details were collected as shown in Fig. 1 . The duration of epilepsy was taken as the time from the onset of first clinical seizure. The patients were grouped into a prodromal, acute, or residual stage based on the disease course at the time of acquisition of FDG PET scan. 9

Behavioral assessment was done by “Achenbach and L. Rescoria” child behavior checklist (CBCL) for 2 to 5 years and the Achenbach CBCL for ages 6 to 16 years. 10 Total CBCL scores were calculated as per the information given by the caregiver who was mostly a parent in our study.

The intelligence quotient (IQ) was assessed using the Binet Kamat test of intelligence (BKT), 11 an Indian version of “Weschler's scale” used since the 1960s. The advantages of this test are low cost, easy to administer, less time consuming, provides a comprehensive score, and is a valid test in terms of standardization. The disadvantage has been in prorating the IQ and mental age which has been taken into account while interpreting the test.

Informed consent was taken from the patient's legal guardian before the FDG PET scans were performed.

### Controls

The control group consisted of children who underwent PET CT for various nonneurologic indications like primary bone malignancy and lymphoma. None of them had any prior history of developmental delay or psychiatric disorders and were not on any medication known to interfere with cerebral metabolism. As ^18^ F FDG PETCT scans were performed for clinical indications, where it was part of clinical management, the need for consent was waived by the ethics committee.

### Fluorodeoxyglucose Positron Emission Tomography Scanning and Analysis

FDG PET scan was performed on GE Discovery 710 whole-body PET-CT scanner. All patients were fasting for at least 6 hours before ^18^ F FDG injection. We injected 5 to 7 MBq/kg body weight (minimum of 74 MBq) and scan was performed at 45 to 60 minutes after injection. PET brain for cases was done for 10 minutes. In Controls, the brain study was a part of the whole-body scan. Usually, the whole body is performed at 1.5 minutes per bed with four to six beds required to cover the whole body. For purpose of the study, we had increased the time of bed in which the brain was to be imaged from 1.5 to 5 minutes. Whole PET-CT scan including brain was finished within 10 minutes. It did not require any additional FDG injection or additional radiation dose from the CT scanner. The difference in the duration of scan in cases (10 minutes) and controls (5 minutes) do not impact the standardized uptake value (SUVmax). 12

The FDG-PET images were reconstructed into axial, coronal, and sagittal cross-sections and analyzed by two experienced nuclear medicine physicians (M.K.S. and K.N.). The results were interpreted qualitatively by visual analysis and quantitatively by estimating the SUV max after drawing a three-dimensional adjustable region of interest (ROI) on various cortical and subcortical structures in all the three planes. The SUVmax represents the maximum FDG uptake seen in any pixel in the given ROI. A total of 17 ROIs were made on cortical and subcortical structures which included right frontal, left frontal, right parietal, left parietal, right mesial temporal, left mesial temporal, right lateral temporal, left lateral temporal, occipital, right caudate, left caudate, right lentiform nuclei, left lentiform nuclei, right thalamus, left thalamus, mid brain, and whole of cerebellum ( Fig. 1 ).

On visual analysis, the abnormality was categorized as “hypometabolic” when the FDG uptake was clearly reduced compared with contralateral region or surrounding cerebral parenchyma and “hypermetabolic” when FDG uptake was more than surrounding cerebral parenchyma. The total number of areas showing hypo- or hypermetabolism was recorded. As the cerebellum is not known to be involved in CSWS, the SUVmax of cerebellum was considered as reference and the ratio of SUVmax of other brain structures was obtained with reference to cerebellar SUVmax. The absolute asymmetry index (AAI) for thalami was calculated by the following formula given by Agarwal et al. 13

AAI for thalami: AAI = (RT − LT) 100 / [(RT + LT) / 2]

### Statistical Analysis

Descriptive statistics were used to summarize demographic variables. Mean and standard deviations were used to analyze continuously scaled variables. Categorical variables were reported using proportions and ratios. Mean and standard deviation (SD) values were calculated for all quantitative variables.

Prevalence and percentages were calculated for the qualitative type of variables. Mean values of SUV of different brain structures were compared across groups using the Student's *t* -test. The relationship of IQ and CBCL scores with the duration of symptoms was assessed by Pearson's correlation. The level of significance was considered as 0.05. SPSS version 24 was used for statistical analysis.

## Results

The study population consists of 28 patients out of which 14 (50%) were cases and 14 (50%) were controls. The mean age of cases was 8.2 ± 2.7 years (range: 4–15 years) and the mean age for controls was 10.1 ± 3.6 years (range: 3–14 years), the difference being statistically insignificant ( *p* = 0.13). The male-to-female ratio for cases was 1.8, while for controls, it was 3.3.

In cases, the majority, that is, 13 of 14 patients (93%), were right-handed and 1 of 14 patient (7%) was left-handed. Nine patients had focal seizures (64%), three patients had generalized seizures (22%), and two patients had focal and generalized seizures (14%). All cases were in the acute stage of disease 9 at the time of the FDG PET scan.

The mean age of onset of Epilepsy in the cases was 4.5 ± 1.7 years (range: 1.5–7 years), while the mean duration of epilepsy was 6 ± 2.8 years (range: 11 months–11.5 years).

Of 14 patients, none of the patients had gross delayed milestones, only one child had mild delayed milestone in the form of delayed speech. Rest all cases had normal development.

The mean IQ of cases using the BKT was 72.4 ± 18.2 (range: 45–104), while mean CBCL score was 37.3 ± 11.8 (range: 16–53). One child was less than 5 years of age whose CBCL score was 31. The various characteristics of the cases are compiled in Table 1 .

**Table 1:** Demographic, clinical profile, and EEG findings in patients with ESES (cases)

Sr. no.	Sex	Age at seizure onset	Age at diagnosis of CSWS	Type of seizures	Regression a/w CSWS	Stage of the disease at time of PET imaging	Intelligence quotient	CBCL score	Frequency of seizures	Current medication
1	F	1.5	4	Foc. + Gen.	BD	Acute	66	38	Infrequent	VPA, CLB
2	M	3.5	7	Gen	–	Acute	73	44	Infrequent	VPA
3	F	6	9	Foc.	BD	Acute	68	45	Infrequent	VPA
4	M	6	9	Foc.	BD	Acute	62	16	Infrequent	VPA, CLB
5	F	5	5	Foc.	BD	Acute	50	53	Infrequent	VPA, steroids
6	F	5	6	Foc.	–	Acute	60	51	Frequent	LEV, VPA, CLB
7	F	6	7	Foc.	–	Acute	72	38	Frequent	VPA, LEV, CLB
8	M	3.5	8	Foc.	–	Acute	45	35	Frequent	VPA, LTG
9	M	3	4	Foc.	BD	Acute	104	31	Frequent	VPA, CLB
10	M	2	6	Foc. + Gen.	Unable to speak	Acute	62	50	Infrequent	VPA
11	M	5.5	8	Foc.	–	Acute	99	25	Infrequent	LTV, LTG, CLB
12	M	7	7	Foc.	BD	Acute	92	28	Infrequent	VPA, LEV
13	M	6	9	Gen.	–	Acute	95	21	Infrequent	VPA, LEV, CLB
14	M	3	6	Gen.	–	Acute	66	41	Frequent	VPA, CLB, steroids

Abbreviations: BD, behavioral disturbance; CBCL, child behavior checklist; CLB, clobazam; CSWS, spike and wave in slow sleep; EEG, electroencephalography; ESES, electrical status epilepticus in sleep; F, female; Foc., focal; Gen., generalized; LAC, lacosamide; LEV, levetiracetam; LTG, lamotrigine; PET, positron emission tomography; S, steroids; VPA, valproic acid.

The behavioral abnormalities which were noticed in all patients during administration of CBCL score predominantly included lack of attention, aggression, and socially inappropriate behavior.

### Intelligence Quotient and Child Behavior Checklist Score in Relation to the Duration of Epilepsy

**Fig. 2:**
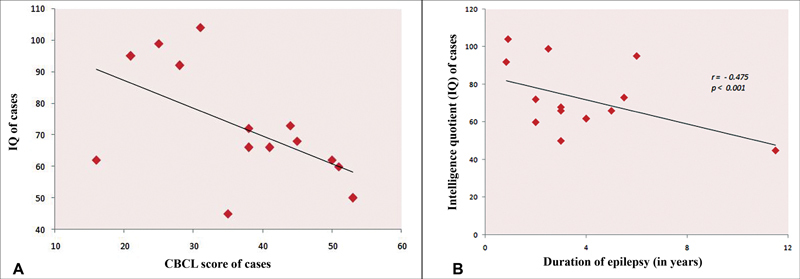
( **A** ) Scatter plot showing Pearson's correlation between IQ and CBCL score of cases. There is negative correlation between IQ and CBCL score, the lower the IQ, the higher the CBCL score and vice versa. ( **B** ) Scatter plot showing Pearson correlation and *p* -value between duration of epilepsy and IQ of cases. There is negative correlation between duration of epilepsy and IQ, that is, lesser the duration of epilepsy, better was the IQ and vice versa. CBCL, child behavior checklist; IQ, intelligence quotient.

There was a moderate negative correlation between duration of epilepsy and IQ of cases ( *r* = −0.475) with a significant *p* -value ( *p* < 0.001) and a moderate negative correlation between IQ and CBCL score of cases ( *r* = −0.55), suggesting more behavioral abnormalities in those with lower IQ which had statistically significant *p* -value ( *p* < 0.001; Figs. 1B and 2A ).

### Electroencephalographic Findings

The EEG data of cases were reviewed to look for possible epileptic focus. There was no evidence of lateralization in the EEG recordings. The maximum amplitude was localized in frontal cortex (4/14 cases), followed by centrotemporal (3/14), centroparietal (3/14), and parietooccipital (3/14) cortex. One patient showed variable focus of maximum amplitude during the period of EEG recording.

### ^18^ F Fluorodeoxyglucose Positron Emission Tomography–Computed Tomography Imaging

#### Visual Analysis

The most striking finding on visual analysis was thalamic hypometabolism. Overall, 12 of 14 patients (85.7%) showed thalamic hypometabolism of which 6 had bilateral diffuse symmetrical involvement, 3 had asymmetrical left thalamic, and 3 had asymmetrical right thalamic hypometabolism. There was also hypometabolism noted in various cortical and subcortical structures. The number of cortical structures showing hypometabolism was 2.3 ± 1.4 (range: 0–4). Similarly, the number of subcortical structures including thalamus showing hypometabolism was 2.2 ± 0.82 (range: 0–4; Fig. 3 ).

**Fig. 3:**
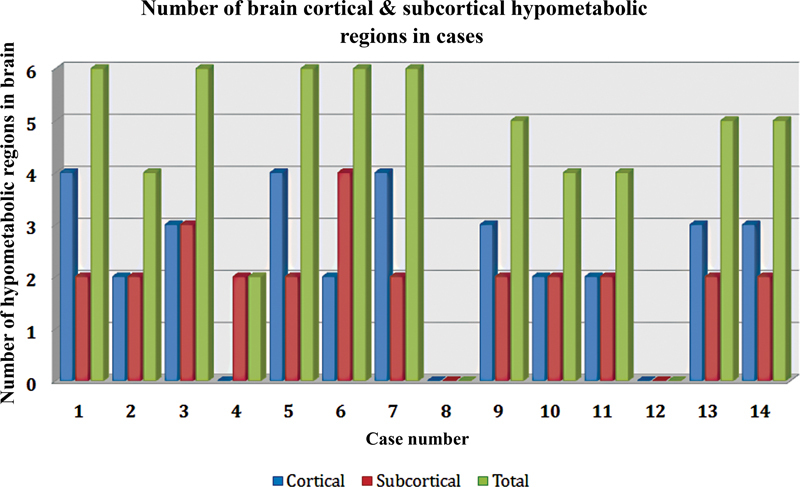
Illustrative diagram showing number of cortical, subcortical and total number of brain regions showing hypometabolism on PET–CT in cases. As shows, patient numbers 8 and 12 did not show any hypometabolism and had normal brain scan. CT, computed tomography; PET, positron emission tomography.

#### Quantitative Analysis

The mean cerebellar SUV uptake in cases was 7.4 ± 2.1 and in controls was 7.5 ± 1.8 which was similar with no statistical significance ( *p* = 0.9). The mean bilateral thalamic SUVmax, AAI for thalami, and ratio of various cortical/subcortical structures (bilateral frontal, parietal, medial and lateral temporal cortices, occipital lobe, caudate, lentiform nucleus, and mid brain) to cerebellum were compared in both cases and control groups with no statistical difference ( Table 2A,B ). Similarly, the mean SUV max in these cortical and subcortical structures were compared between cases and controls but did not show any statistical significance ( Table 2C ).

**Table 2:** SUVmax in various brain regions in cases and controls on FDG PET-CT study after drawing ROIs: (A) on thalamus, (B) cortical and subcortical structures in reference of cerebellum, and (C) absolute SUVmax of cortical and subcortical structures

** (A) SUVmax of thalami and absolute asymmetry index (Agarwal et al 12 ) in cases and controls. The SUVmax represents the maximum value of FDG uptake seen in the region of interest **			
**Parameters**	**Cases**	**Controls**	***p*** **-Value**
Right thalamus	8.14	8.60	0.53
Left thalamus	8.09	8.74	0.65
Absolute asymmetry index	1.10	−1.58	0.42
**(B) Ratio of SUVmax of cortical and subcortical structure with respect to cerebellum in cases and controls. The SUVmax represents the maximum value of FDG uptake seen in the region of interest**			
Right mesial temporal	0.932	0.924	0.913
Left mesial temporal	0.947	0.914	0.703
Right lateral temporal	1.174	1.165	0.908
Left lateral temporal	1.18	1.143	0.534
Right frontal	1.479	1.368	0.273
Left frontal	1.496	1.375	0.223
Right parietal	1.418	1.339	0.409
Left parietal	1.462	1.317	0.175
Occipital	1.503	1.46	0.72
Mid brain	0.758	1.252	0.363
Right caudate	1.293	1.104	0.023
Left caudate	1.287	1.125	0.04
Right lentiform nucleus	1.45	1.21	0.02
Left lentiform nucleus	1.46	1.20	0.03
**(C) Absolute SUVmax of cortical and subcortical structure in cases and Controls. The SUVmax represents the maximum value of FDG uptake seen in the region of interest**			
Right mesial temporal	6.651	6.918	0.635
Left mesial temporal	6.674	6.89	0.715
Right lateral temporal	8.454	8.709	0.703
Left lateral temporal	8.614	8.605	0.99
Right frontal	10.69	10.32	0.7
Left frontal	10.83	10.36	0.625
Right parietal	10.21	10.12	0.409
Left parietal	10.51	9.995	0.921
Occipital	10.87	11.0	0.91
Mid brain	5.426	5.352	0.866
Right caudate	9.721	8.35	0.143
Left caudate	9.689	8.539	0.251
Right lentiform nucleus	10.44	9.768	0.462
Left lentiform nucleus	10.42	9.657	0.40

Abbreviations: FDG, fluorodeoxyglucose; PET, positron emission tomography; ROI, region of interest; SUVmax, standardized uptake value.

The ratio of right caudate nucleus to cerebellum SUVmax values in cases and controls was 1.29 ± 0.24 and 1.14 ± 0.14, respectively, which showed statistical significance ( *p* = 0.023). Ratios of left caudate nucleus to cerebellum mean SUVmax values in cases and controls were 1.28 ± 0.25 and 1.10 ± 0.14, respectively, which was statistically significant ( *p* = 0.02). Similarly, the ratio of right lentiform nucleus to cerebellum mean SUV values in cases and controls was 1.45 ± 0.36 and 1.21 ± 0.12, respectively, which was statistically significant ( *p* = 0.02). Ratios of the left lentiform nucleus to cerebellum mean SUV values in cases and controls were 1.46 ± 0.38 and 1.20 ± 0.12, respectively, which was statistically significant ( *p* = 0.03). This is a new finding in our study, suggesting involvement of striatothalamic tract.

### Correlation of Electroencephalographic and Fluorodeoxyglucose Positron Emission Tomography–Computed Tomography Findings

The total regions of cortical and subcortical hypometabolisms, as depicted in Fig. 3 , was correlated with EEG findings in the cases and showed a weak correlation ( *r* = 0.339) and nonsignificant *p* -value ( *p* = 0.096). Overall, 6 of 14 patients (43%) showed hypometabolism in the same cortical areas where there was maximum amplitude of interictal spikes on EEG. Other patients did not show similar concordance.

### Correlation of Fluorodeoxyglucose Positron Emission Tomography Findings with Duration of Epilepsy

**Fig. 4:**
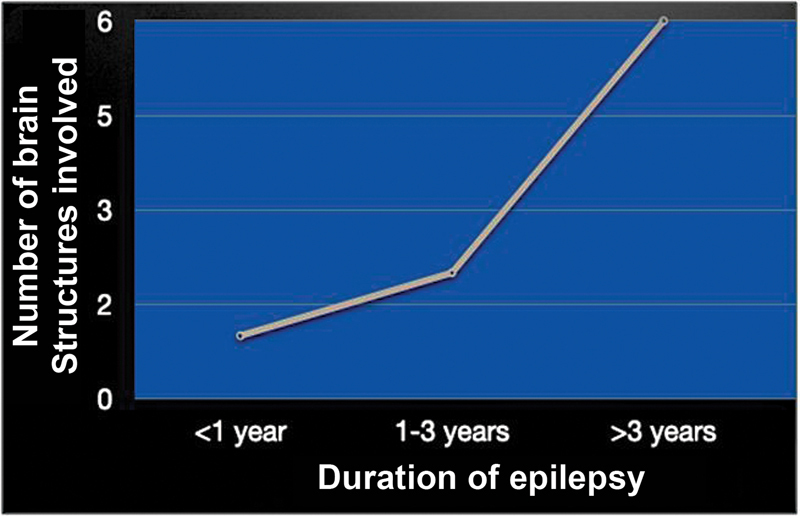
Number of cortical and sub cortical structures involved with respect to duration of ESES. As the duration of ESES increased, the number of cortical and sub cortical structures showing hypometabolism increased. ESES, electrical status epilepticus in sleep.

We found a strong correlation between duration of epilepsy and the total number of cortical and subcortical regions that showed hypometabolism ( Fig. 4 ). This suggests that the longer the duration of epilepsy/ESES, the more are the number of cortical and subcortical hypometabolic regions in the brain.

### Correlation of Child Behavior Checklist Score, Intelligence Quotient with Fluorodeoxyglucose Positron Emission Tomography–Computed Tomography Findings

**Fig. 5:**
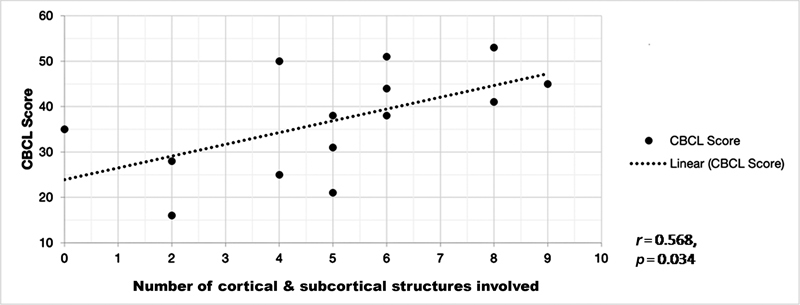
Correlation graph showing correlation between CBCL score and number of cortical and subcortical regions showing hypometabolism on FDG PET study. There is weak positive correlation suggesting more the regions of brain hypometabolism, higher the behavioral abnormalities. CBCL, child behavior checklist; FDG, fluorodeoxyglucose.

**Fig. 6:**
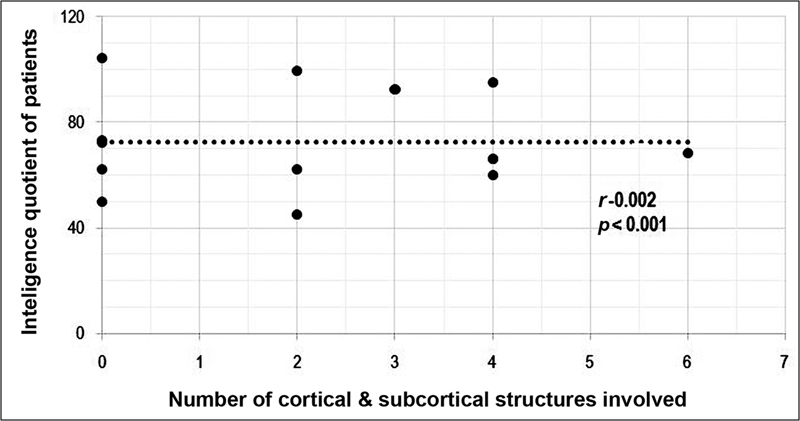
Correlation graph showing correlation between IQ and number of cortical and subcortical regions showing hypometabolism on FDG PET study. There is weak negative correlation suggesting more the area of brain hypometabolism, lower is the intelligence quotient of the child. CT, computed tomography; IQ, intelligence quotient; PET, positron emission tomography.

**Fig. 7:**
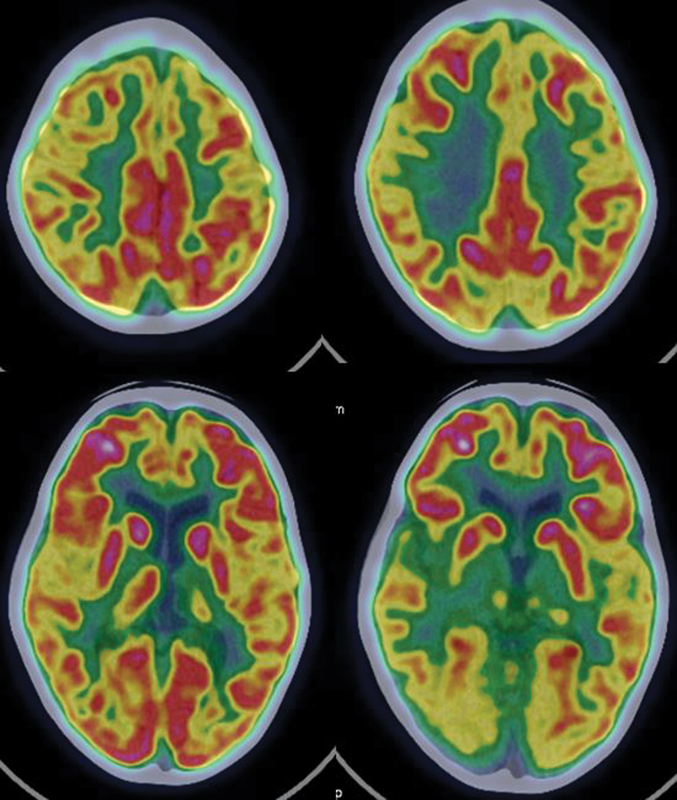
A 7-year-old left-handed girl, with positive family history, normal milestones presented with focal and generalized seizures in sleep since 1.5 years of age associated with significant behavioral disturbances. Her IQ was 66 and CBCL score was 38. There were no focal neurological deficits. EEG showed left parietooccipital predominance during the ESES. PET-CT showed hypometabolism in left thalamus, bilateral anterior cingulate and lateral temporal cortices. The Right thalamus showed SUVmax of 8.2, but visually, it showed reduced FDG uptake in ventral group and pulvinar group of nuclei and relatively normal FDG uptake in anterior and dorsal median group of nuclei. Rest of the cortical and subcortical structures showed normal metabolism. CBCL, child behavior checklist; CT, computed tomography; ESES, electrical status epilepticus in sleep; FDG, fluorodeoxyglucose; IQ, intelligence quotient; PET, positron emission tomography; SUVmax, standardized uptake value.

**Fig. 8:**
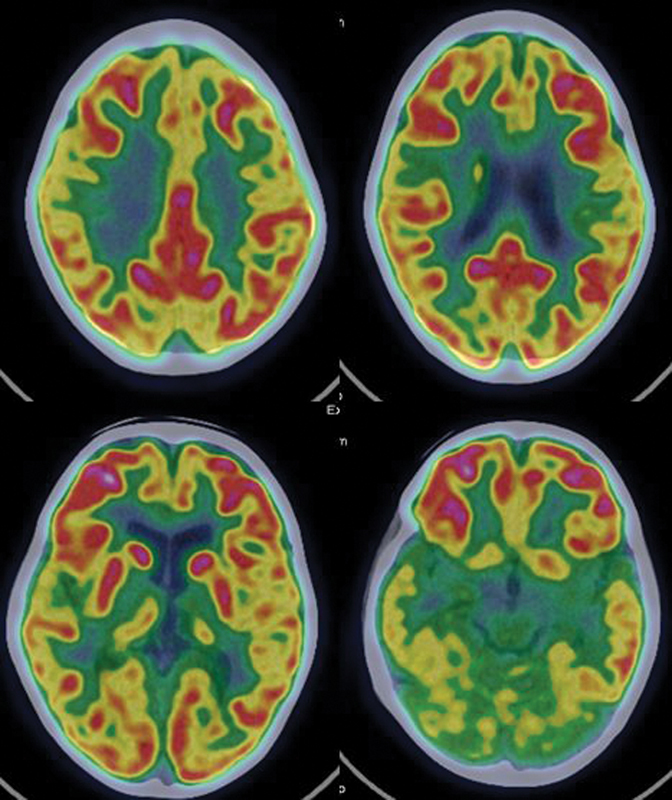
A 10-year-old right-handed boy, with normal milestones presented with focal seizures in sleep since 6 years of age associated with behavioral disturbances and poor scholastic performance. His IQ was 62 and CBCL score was 16. There were no focal neurological deficits. EEG showed left centroparietal predominance during the ESES. PET-CT showed hypometabolism in both thalami (left > right) with relatively normal metabolism in rest of the brain parenchyma. CBCL, child behavior checklist; CT, computed tomography; EEG, electroencephalography; ESES, electrical status epilepticus in sleep; IQ, intelligence quotient; PET, positron emission tomography.

We correlated the total number of cortical and subcortical regions showing FDG hypometabolism, as seen in Fig. 2 , with the IQ and CBCL score. There was a moderately positive correlation between total number of hypometabolic regions in brain and CBCL score ( *r* = 0.568) which showed statistical significance ( *p* = 0.034), suggesting that the brain hypometabolism is producing the neuropsychological deficits ( Fig. 5 ). There was no significant correlation between the IQ and number of brain's hypometabolic areas ( Fig. 6 ), as shown in two illustrative cases ( Figs. 7 and 8 ).

### Fluorodeoxyglucose Positron Emission Tomography Findings in Relation to Treatment

Twelve out of 14 patients with ESES were on antiepileptic drugs before the PET-CT imaging. In two patients, who received early medical attention and were given steroids, their FDG PET study of the brain was normal.

## Discussion

There are few studies that compared the clinical profile of the ESES patients with brain metabolism on ^18^ F FDG PET–CT imaging. We studied PET abnormalities in ESES patients and correlated it to clinical and neuropsychological status.

The male-to-female ratio with ESES in our study was 1.8:1 which is slightly higher than seen in other studies like Yilmaz et al, 14 while the mean age of diagnosis of ESES in our study was 8.2 ± 2.7 years which is similar to that seen by Agarwal et al. 13

Scholtes et al 15 reported that the epileptic disturbance occurring early in pediatric age group results in greater degree of functional deficit. Our study substantiates this view as we found that with increase in duration of epilepsy, the ESES children tended to have lower IQ. Another interesting finding in our study was that as duration of disease increased, more brain areas were affected on FDG brain study, resulting in behavioral abnormalities and lower IQ status. To authors knowledge, this is the first study to show direct comparison of FDG brain study with CBCL score and IQ of the patients.

Majority of patients with CSWS may have a normal brain MRI without noticeable thalamic abnormalities, 9 14 16 similar to our cohort where all 14 cases had normal MRI brain. Thalamic abnormality in ESES patients was first established by Incorpora et al 17 and later studies also proved that early thalamic injury might lead to CSWS over some time. 16 18 19 The presence of genetic mutation or disturbances in the function of ion channels may distort the functional capacity of thalamus, not detected by routine MRI study. FDG-PET study helps in detecting this functional impairment and is thus more sensitive compared with routine MRI. 20

There is a few literature on use of ^18^ F FDG PET–CT imaging in ESES patients. Agarwal et al 13 showed that functional abnormalities are common in ESES patients with normal structural MRI. Further, the study showed that AAI of thalami can be an important tool in the investigation of ESES patients. Our study demonstrated thalamic hypometabolism in 12 out of 14 cases on qualitative visual analysis, though it did not find any difference quantitatively in AAI of thalami in patients and controls. This can be explained by the fact that only a part of thalamus representing few nuclei could be abnormal and not the whole thalamus ( Fig. 7 ). First, as ROI is drawn on the whole thalamus and not only on the hypometabolic part of the thalamus but also there is an automatic quantitative estimation of SUVmax in both thalamus which represents the maximum FDG uptake in any pixel in the ROI. This results in false increase in the SUVmax values which is required for estimation of AAI, so that the AAI index did not reveal any statistical difference. Second, the number of cases in our study is small, and, third, the controls are not true controls but pseudocontrols, that is, the children are taken as controls have some other oncologic and no-oncological disease for which PET-CT was done unlike Agarwal et al who had true controls.

There is transient thalamic activation during CSWS as seen by EEG–functional MRI (fMRI) studies, 21 suggesting that thalamus potentially plays a role in propagation and diffusion of epileptic activity generated in the cortex to other brain areas. Our study establishes that functional thalamic abnormalities reflected by low FDG uptake, either unilateral or bilateral, were seen in the majority of patients with ESES, suggesting that abnormal thalamic functions are closely linked to ESES.

The new finding in our study was hypermetabolism of basal ganglion involving both caudate and lentiform nuclei compared with cerebellum. All these patients also had thalamic hypometabolism. We hypothesize that the basal ganglionic hypermetabolism is secondary to thalamic involvement. The basal ganglionic–thalamic neuronal network occurs through multiple parallel closed and open loops. 22 23 This has been well studied in multiple other neurological conditions like Parkinson's disease. However, it has not been explored in epilepsy so far.

To the author's knowledge, this is the first study that had tried to correlate clinical and neuropsychological status in ESES patients with brain metabolism seen on FDG PET–CT study. The strength of the study is the uniform cohort, that is, all cases with similar clinical and radiological profiles were included. However, the cross-sectional design of the study does not establish direct temporal relationship between ESES and thalamic and focal cortical/subcortical hypometabolism, although it substantiates the previously established hypothesis. Our control group was not a true control group but essentially “pseudocontrols” which might be a major limiting factor. Moreover, the lack of quantitative data about FDG-PET uptake in a normally developing brain remains a limitation in most pediatric PET studies due to ethical and logistical constraints of performing a scan in a completely normal healthy child. The authors think that further multicentric studies with larger sample size, serial imaging studies, and normal age-matched controls would give better look into the proposed pathophysiology of ESES.

The findings of normal PET imaging in two cases, even though a very small number, who received steroids, suggest that early intervention can resolve the cerebral metabolic defects.

## Conclusion

Our study demonstrates functional impairment of cerebral cortical, basal ganglia, and thalamic hypometabolism in a cohort of ESES patients with normal structural MRI. There was a moderate correlation of extent and pattern of cerebral hypometabolism with the neuropsychological status of the child, EEG findings, and duration of epilepsy. Further long-term studies are required to establish this correlation.
